# Inferred ancestry of scytonemin biosynthesis proteins in cyanobacteria indicates a response to Paleoproterozoic oxygenation

**DOI:** 10.1111/gbi.12514

**Published:** 2022-07-18

**Authors:** Erik Tamre, Gregory P. Fournier

**Affiliations:** ^1^ Department of Earth, Atmospheric and Planetary Sciences Massachusetts Institute of Technology Cambridge Massachusetts USA

**Keywords:** Bayesian phylogenetics, cyanobacteria, Paleoproterozoic, photoprotection, scytonemin, UVA radiation

## Abstract

Protection from radiation damage is an important adaptation for phototrophic microbes. Living in surface, shallow water, and peritidal environments, cyanobacteria are especially exposed to long‐wavelength ultraviolet (UVA) radiation. Several groups of cyanobacteria within these environments are protected from UVA damage by the production of the pigment scytonemin. Paleontological evidence of cyanobacteria in UVA‐exposed environments from the Proterozoic, and possibly as early as the Archaean, suggests a long evolutionary history of radiation protection within this group. We show that phylogenetic analyses of enzymes in the scytonemin biosynthesis pathway support this hypothesis and reveal a deep history of vertical inheritance of this pathway within extant cyanobacterial diversity. Referencing this phylogeny to cyanobacterial molecular clocks suggests that scytonemin production likely appeared during the early Proterozoic, soon after the Great Oxygenation Event. This timing is consistent with an adaptive scenario for the evolution of scytonemin production, wherein the threat of UVA‐generated reactive oxygen species becomes significantly greater once molecular oxygen is more pervasive across photosynthetic environments.

## 1 INTRODUCTION

Throughout history of life on Earth, organisms have found ways to alleviate the harmful effects of ultraviolet (UV) radiation on living cells and their genetic material. The efforts to escape harm can take three basic forms: responding to UV radiation by moving away from it, blocking it before it can do damage, or repairing the damage after the fact. Most organisms have some capacity to heal radiation damage that has already occurred, as exemplified by widespread DNA repair mechanisms (Rastogi et al., 2010). Some organisms have also developed complex and specific responses based on the limiting access of UV radiation to their cells. Phototrophic microbes such as cyanobacteria are exposed to potential UV damage due to their dependence on solar radiation in environments with high UV flux.

### 1.1 History of UV exposure on earth

The amount and kind of UV radiation incident on the Earth's surface has varied through its history, depending on both stellar evolution and atmospheric changes (Cnossen et al., 2007) (Segura et al., 2003). The most substantial change in surface UV incidence occurred with the formation of the ozone layer. Ozone is produced in the upper atmosphere by UV‐catalyzed radicalization of dioxygen, and the expected column depth (“thickness”) of the ozone layer can be estimated directly from atmospheric oxygen concentrations (Segura et al., 2003). The history of oxygen in the Earth's atmosphere remains a topic of considerable debate, but it is now clear that global oxygen concentration first persistently rose above $10^{-5}$ of the present atmospheric level (PAL) around 2.3–2.4 Ga, during the “Great Oxygenation Event” (GOE). At that time, mass‐independent sulfur fractionation, possible in an atmosphere with oxygen levels below $10^{-5}$ PAL, ceased irreversibly (Reinhard et al., 2013). After the GOE, it remains unclear how far above the $10^{-5}$ PAL threshold oxygen levels were: estimates range from $10^{-4}$ to $10^{-1}$ PAL for the subsequent period of almost 2 billion years, until oxygen levels finally began to approach modern values starting around 600 Ma (Lyons et al., 2014).

The UV‐protective effect of the ozone layer increases non‐linearly with atmospheric oxygen concentration: while oxygen levels below $10^{-5}$ PAL have little impact on the UV radiation reaching the ground, $10^{-2}$ PAL results in an ozone layer that filters out almost all UVC (<280 nm) and most UVB (280–315 nm) radiation; a further increase to 1 PAL changes relatively little (Segura et al., 2003). Thus, we can expect the overall UV exposure to have been harsh before the GOE and similar to the present during the Phanerozoic, while uncertainty reigns during the Proterozoic. UVA (320–400 nm) is not much impacted by the ozone layer at any concentration (Segura et al., 2003). However, while the amount of UVA reaching the ground has likely not changed significantly since the Archaean, its impact on organisms has: UVA only becomes significantly injurious to most cells in the presence of oxygen, producing reactive oxygen radicals and increasing oxidative stress (Garcia‐Pichel, 1998). Thus, counterintuitively, UVA protection became more important *after* the GOE and the inception of the ozone layer, even as the incidence and impact of shorter‐wavelength UV decreased. It is also possible that some of the deleterious effect of UVA would have been experienced by cyanobacteria locally producing oxygen before the GOE. In such a case, the localized impact of UVA would have accompanied the effects of a much greater potential exposure to UVB and UVC at the time.

Of all the groups of organisms through Earth history, it is likely that cyanobacteria have felt the effect of UV radiation most keenly. They are phototrophs, which sets up a basic trade‐off: they need to get enough photosynthetically active radiation, but this also exposes them to the harm of UV. They were likely present under the much harsher radiation conditions of the past: stromatolitic structures of size and shape analogous to modern cyanobacterial mats have been found in rocks as old as 3.0 Ga (Bosak et al., 2013). Furthermore, from early on many cyanobacteria inhabited relatively UV‐exposed environments: while it is harder to make broad statements about their environmental distribution in the late Archaean, evidence of cyanobacteria has been reported in rocks from peritidal settings spanning much of the Proterozoic (Knoll, 2015). Microfossils and microbially induced sedimentary structures from subaerial communities analogous to those dominated by cyanobacteria today have also been observed as early as Mesoproterozoic (Beraldi‐Campesi et al., 2014). As such, cyanobacteria have dealt with the challenges of UV radiation for a long time, and their history of photoprotection offers a deep insight into the changing habitability conditions in these exposed environments.

### 1.2 UV‐protective strategies

UV radiation can cause damage to cellular components directly as well as indirectly by creating reactive oxygen radicals; organisms across the tree of life use specialized proteins to regularly check the genome for UV damage and initiate repair pathways or even apoptosis in response (Rastogi et al., 2010). If UV radiation creates oxygen radicals, enzymes in detoxification pathways such as catalase, peroxidase, and superoxide dismutase act to remove them (Xie et al., 2009); some antioxidants such as vitamin E also react with the reactive oxygen species themselves (He et al., 2017). In cyanobacteria, carotenoids commonly perform antioxidant activity and experiments have shown an increase in their cellular quota in response to UV radiation (Ehling‐Schulz & Scherer, 1999).

Photoprotective pigments prevent such damage by safely absorbing harmful radiation. Flavonoids commonly play this role in plants (Agati & Tattini, 2010), while mycosporine‐like amino acids (MAAs) fulfil this function in diverse groups across the tree of life, including bacteria, eukaryotic algae, fungi, and even animals (Sinha et al., 2007); they are also widely found in Cyanobacteria (Llewellyn et al., 2020). In addition, some cyanobacteria produce the UV‐protective pigment scytonemin, which is unique to this group (Ferreira & Garcia‐Pichel, 2016). While scytonemin offers some protection against short‐wavelength UV (Dillon & Castenholz, 1999), its in vivo absorption maximum is in the UVA range at 372 nm (Castenholz & Garcia‐Pichel, 2012). It has been shown that UVA can significantly damage cyanobacterial cells in the absence of scytonemin: for example, a culture of *Cylindrospermopsis raciborskii* lacking scytonemin significantly suffered in cell concentration as well as viability when radiated with UVA for 6 h (Noyma et al., 2015).

While different MAAs have their absorption maxima in various parts of the UVB and UVA range and some could partially substitute for scytonemin, the ones commonly present in cyanobacteria do not absorb significantly at wavelengths longer than 340 nm (Garcia‐Pichel & Castenholz, 1993). Thus, scytonemin is responsible for most of the protection in the UVA range in Cyanobacteria. Experimental evidence from *Lyngbya* supports this delineation: MAA synthesis increases upon irradiation with UVB, while scytonemin synthesis increases more with UVA (Rastogi & Incharoensakdi, 2013). Another important difference between the two pigments is their placement: scytonemin is discharged into the extracellular matrix and can absorb radiation before it ever reaches the cells, while MAAs are generally intracellular compounds (Garcia‐Pichel & Castenholz, 1993).

### 1.3 Genetics of scytonemin biosynthesis

Genes for scytonemin biosynthesis are usually organized as a single operon, containing (1) *scy* genes primarily responsible for core enzymatic steps of the biosynthesis, (2) *ebo* genes that transport an intermediate synthetic product into the periplasm for final assembly, and (3) additional copies of genes also present elsewhere in the genome that partake in making precursors—such as tryptophan (Klicki et al., 2018). The *ebo* genes are present in many other bacterial phyla in addition to Cyanobacteria and even in the plastids of eustigmatophyte algae (hence the “**e**ustigmatophyte‐**b**acterial **o**peron”, *ebo*) (Yurchenko et al., 2016). Thus, their function is unlikely to be specific to scytonemin biosynthesis, and they might play a more generalized role in metabolite transport (Klicki et al., 2018).

Of the six Scy proteins (A–F, see Table 1), ScyD and ScyF are not essential for making scytonemin, and ScyD is a duplicate of ScyE (Ferreira & Garcia‐Pichel, 2016), presenting a more complex evolutionary history to resolve and interpret. Therefore, we focus on the signature biosynthetic enzymes ScyC, ScyB, and ScyA, which together provide a diagnostic genomic signal for scytonemin biosynthesis capacity.

**TABLE 1:** Overview of scytonemin biosynthesis proteins ScyA‐F

	ScyA	ScyB	ScyC	ScyD	ScyE	ScyF
Requirement (Ferreira and Garcia‐Pichel 2016)	Essential			Not needed, no identified use	Essential	Helpful, but not essential
Function (Ferreira & Garcia‐Pichel 2016; Klicki et al. 2018)	Enzymes in early biosynthesis (cytoplasm)			Unknown, duplicate of ScyE	Dimerization enzyme (periplasm)	Biosynthetic adjuvant
Length (AA) in *N. commune*	628	353	323	428	457	393

Previous investigations of the evolutionary history of scytonemin biosynthesis have already suggested a generally ancient origin, based on its broad taxonomic distribution within Cyanobacteria (Garcia‐Pichel, 1998) and data from limited phylogenetic analyses (Sorrels et al., 2009). A prior effort toward quantitatively timing the evolutionary history of scytonemin focused on directly time‐calibrating phylogenies of the protein families included in the scytonemin operon that make precursors for its synthesis (Garcia‐Pichel et al., 2019). In our study, we generate phylogenies of diagnostic scytonemin biosynthesis enzymes ScyC, ScyB, and ScyA, and we map their origin and evolutionary history to cyanobacterial species trees dated using fossil‐calibrated molecular clocks.

Using divergence times from published molecular clocks of cyanobacterial species trees takes advantage of datasets with a much greater site and taxon sampling than single gene trees can provide. Additionally, dated species trees can include informative calibrations that fall outside of the diversity present in the gene tree. In addition to improved timing estimates for the evolution of scytonemin, these constraints allow us to correlate the phylogeny of scytonemin biosynthesis genes to changes in planetary environment and cyanobacterial ecology. Thus, this phylogenetic approach provides an improved understanding of the conditions and selective pressures—especially regarding exposure and UVA irradiation—that Cyanobacteria faced early in their evolution, during a time where the geologic and geochemical record alone provides scant clues.

## 2 METHODS

### 2.1 Protein sequence collection

To gather Scy protein amino acid sequences for the analysis, we searched the NCBI non‐redundant protein database (National Library of Medicine [US] & National Center for Biotechnology Information, 1988) with BLAST (Altschul et al., 1990) using Scy protein copies in *Nostoc commune* as queries (accession numbers WP_109008285, WP_109008284, and WP_109008282 for ScyC, ScyB, and ScyA, respectively). A BLAST search for ScyC using default parameters returned 71 closely related cyanobacterial sequences and one slightly more distant sequence from *Methylocaldum marinum*, a methane‐oxidizing gammaproteobacterium (current E‐value 10^−117^, identity 53% at 96% query cover). All other hits had an E‐value above 1 and were not included as potential homologs.

Other Scy proteins did not present as such isolated clusters in the sequence space. Thus, similarity cutoffs were more subjectively established where hits became generally non‐cyanobacterial (57% identity at 97% query cover and current E‐value 10^−133^ for ScyB; 54% identity at 89% query cover and negligible E‐value for ScyA). Especially for ScyB, the chosen cutoff also corresponds to a substantial decrease in sequence similarity. Altogether, 81 sequences were selected for ScyB and 157 for ScyA, with the higher number reflecting the presence of two copies in most taxa. *Methylocaldum marinum* appears in each case as the closest non‐cyanobacterial sequence to the query.

90% of genomes with ScyC homologs (the most restrictive of the three sets) contained detected homologs to all three Scy proteins, with incomplete sets of Scy proteins appearing in some Nostocales as well as unclassified Cyanobacteria.

### 2.2 16S rRNA sequence collection

To construct a reference species tree including the taxa on the protein trees, pre‐aligned 16S rRNA sequences from these taxa were collected from the SILVA database (Quast et al., 2012). Each taxon that appears on all three protein trees is included, with the following exceptions: *Pleurocapsa* sp. CCALA 161, *Scytonema hofmanni* UTEX B 1581, and *Chlorogloeopsis* sp. Cgs‐089 were absent from the database and omitted, while *Aphanothece hegewaldii* CCALA 016 was absent and replaced with the closely related strain *A. hegewaldii* SAG 253.80. To better resolve basal relationships between Cyanobacteria in the tree, *Gloeobacter violaceus* CCALA 980 (lacking Scy proteins) was added as a closer outgroup than *M. marinum*. Where taxa had multiple slightly divergent copies of 16S rRNA, the sequence with the highest number of copies was chosen as the representative sequence.

### 2.3 Protein sequence alignment

Multiple sequence alignment of the protein sequences was performed using mafft 7.245 (Katoh & Standley, 2013) with the automatic choice of alignment algorithm (“mafft ‐‐auto”) selecting L‐INS‐i, an accurate iterative refinement approach using local pairwise alignment information.

### 2.4 Protein tree search

We used ProtTest (Darriba et al., 2011) to determine the optimal evolutionary model for the protein alignment data. The substitution model was chosen based on the Bayesian information criterion (BIC), which identified the best‐fitting model as LG (Le & Gascuel, 2008) with four gamma‐distributed site rates and empirical amino acid frequencies (LG + G4 + F). We did not assume any invariant sites in the alignment. With these model choices, we built Bayesian phylogenetic trees using PhyloBayes 4.1 (Lartillot & Philippe, 2004) (Lartillot & Philippe, 2006) (Lartillot et al., 2007). Convergence between MCMC chains was confirmed using TRACECOMP (requiring maximum discrepancy <0.1 and minimum effective size >100) and BPCOMP (requiring maxdiff <0.15). Each chain sampled ~8000, ~6000, and ~20,000 trees for ScyC, ScyB, and ScyA, respectively, including a 20% burn‐in assumed for convergence tests and posterior sampling.

### 2.5 16S rRNA tree search

GTR was chosen as the substitution model for the 16S rRNA tree search as the most general model available. We used four gamma‐distributed site rates and empirical frequencies (GTR + G4 + F). We did not assume any invariant sites in the alignment. We proceeded similarly to the protein trees, building a Bayesian phylogenetic tree using PhyloBayes 4.1 (Lartillot et al., 2007; Lartillot & Philippe, 2004, 2006). Convergence was confirmed using TRACECOMP (requiring maximum discrepancy <0.3 and minimum effective size >50) and BPCOMP (requiring maxdiff <0.3), with each chain sampling 1600 trees in total.

### 2.6 Protein tree‐species tree reconciliation

To quantitatively confirm whether Scy protein trees reflected vertical inheritance of these proteins, we performed protein tree‐species tree reconciliation between each of the Scy trees and the 16S tree using Notung 2.9 (Stolzer et al., 2012). We used the default costs implemented in the program: duplication 1.5, transfer 3.0, and loss 1.0. The inference of duplications, transfers, and losses was successful for ScyC and ScyB. No temporally feasible solutions were found for ScyA.

## 3 RESULTS

### 3.1 ScyC phylogeny

The phylogeny of ScyC provides the most stringent overview of potentially scytonemin‐capable organisms, as it includes the fewest number of taxa: every taxon that has a ScyC homolog also appears in the ScyA and ScyB trees, but there are some taxa with ScyA and ScyB that do not appear to have a ScyC homolog and thus should not be able to produce scytonemin. Furthermore, each taxon has only one copy of ScyC.

**FIGURE 1:**
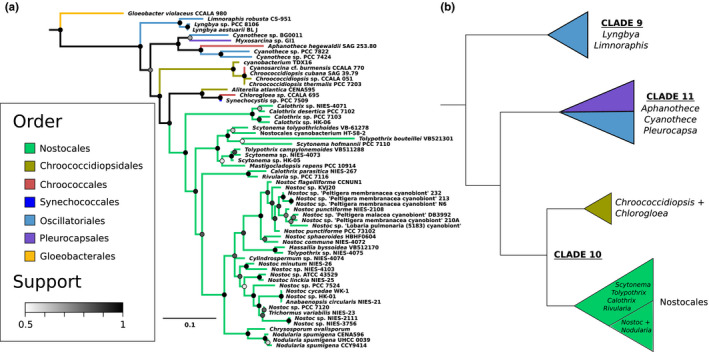
(a) Species tree of all scytonemin‐capable taxa included in this study, based on their 16S rRNA sequences. (b) A simplified species tree of the taxa, summarizing existing literature and illustrating the basic clade structure that should be present in the protein trees to infer their vertical inheritance (notwithstanding phylogenetic uncertainty and possible non‐vertical histories in shallow groups). Clade‐specific colors correspond to the 16S tree (Figure 1b) and the Scy protein trees (Figures 2, 3, 4). The summary tree is based on Moore et al. (2019), Schirrmeister et al. (2011), and Tomitani et al. (2006); clade numbers refer to Moore et al. (2019). The *Cyanothece* on the tree have since been re‐classified as *Gloeothece* (Sayers et al., 2008). Both clades 9 and 11 contain members of Oscillatoriales (cyan), as it is not a monophyletic group.

**FIGURE 2:**
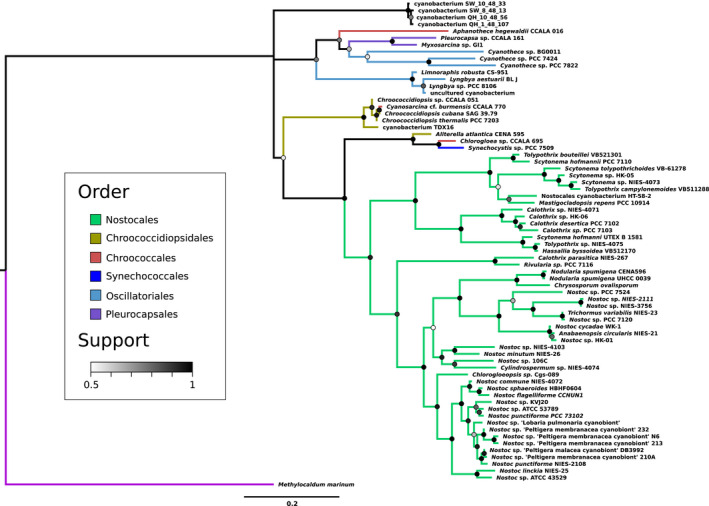
Bayesian phylogenetic tree based on scytonemin biosynthesis protein ScyC sequences. Branches are colored according to taxonomic order as provided by the NCBI taxonomy database (Sayers et al., 2008); note that orders are not always monophyletic. Support of bipartitions in the consensus tree is shown by node color. Branch lengths indicate the average number of substitutions per site; see scale bar at the bottom. One divergent partial copy in *Nostoc* sp. NIES‐4103 has been removed for clarity.

We compare the ScyC phylogeny to a 16S rRNA tree with the same taxon sampling (Figure 1a) as well as previously published species trees (Moore et al., 2019; Schirrmeister et al., 2011; Tomitani et al., 2006), summarized in Figure 1b. In general, the ScyC phylogeny (Figure 2) closely resembles the species trees for a clade of Cyanobacteria including the order Nostocales and some closely related groups. This suggests that scytonemin‐producing cyanobacteria obtained this trait largely through vertical inheritance, from a common cyanobacterial ancestor which could produce scytonemin. Departures from the species tree topology tend to be shallow in clade depth, and represent either relatively recent horizontal gene transfer events between cyanobacterial groups, or a lack of resolution within the gene tree.

The outgroup to *Nostoc* and its close relatives on the species tree is *Rivularia* sp. 7116 (Moore et al., 2019), which is the outgroup on the ScyC tree as well. Genus *Nostoc* itself does not appear as monophyletic, but this agrees with previously published trees (Tomitani et al., 2006) as well as the 16S tree. The earliest‐branching group within Nostocales on the species tree comprises taxa from *Calothrix*, *Tolypothrix*, and *Scytonema*—also in agreement with the ScyC tree. However, there are some conspicuous absences within Nostocales on the ScyC tree: for example, *Fischerella* species do not appear to have ScyC. This likely reflects a gene loss event, corresponding to loss of ability to produce scytonemin.

The expected outgroup to Nostocales in Moore et al. (2019) is a clade consisting of *Chlorogloea* CCALA695 sister to a closely related cluster of *Cyanosarcina* CCALA770 and some *Chroococcidiopsis*. The ScyC tree departs from this only slightly, placing *Chlorogloea* CCALA695 as sister to Nostocales and the *Cyanosarcina* + *Chroococcidiopsis* cluster as the outgroup. The support for grouping *Cyanosarcina* + *Chroococcidiopsis* with Nostocales + *Chlorogloea* is also relatively low, with a posterior probability of 55%. However, this grouping is in complete agreement with the 16S tree.

The next outgroup beyond *Chroococcidiopsis* includes taxa from Clade 11 as defined in Moore et al. (2019); *Chroococcidiopsis* completes Clade 10 according to this labelling, which we will also use hereafter. Like in Clade 10, there are significant losses among the Clade 11 taxa, as representatives of *Stanieria*, *Spirulina*, *Coleofasciculus*, and *Moorea* on the species tree do not seem to have ScyC. The Clade 11 representatives that do appear on the ScyC tree form a clade. The shallow relationships differ slightly from past studies and the 16S tree—indeed, internal supports within this group fall almost down to 50%—but the support for the clade as a whole is 76%.

Finally, as sister to Clade 11 on the ScyC tree, we find a small group including *Lyngbya* and *Limnoraphis*. Phylogenetic analyses of their placement in the species tree suggest that they belong to Clade 9 with *Phormidium*, *Arthrospira*, and *Trichodesmium* (Komarek et al., 2013; Kothari et al., 2013). This is a departure from previous species trees and the 16S tree, which both suggest a placement basal to all other taxa in the ScyC tree. In other words, the root should fall slightly differently: between Clade 9 taxa and the other Cyanobacteria on tree, rather than on the adjoining branch (see section on rooting below).

The automated protein tree‐species tree reconciliation (Figure S1 for ScyC) supports these inferences, identifying the placement of *Lyngbya* and *Limnoraphis* together with the Clade 11 taxa as the deepest suggestion of a non‐vertical inheritance event. Other identified events are relatively shallow, and the reconciliation is consistent with the suggestion that ScyC has generally been inherited vertically among the scytonemin clade.

### 3.2 ScyB phylogeny

**FIGURE 3:**
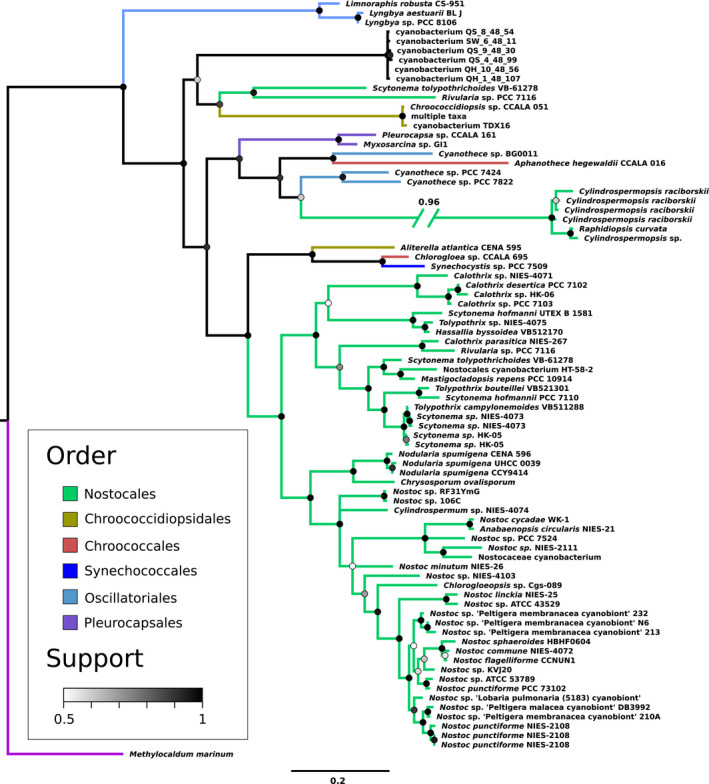
Bayesian phylogenetic tree based on scytonemin biosynthesis protein ScyB sequences. Branches are colored according to taxonomic order as provided by the NCBI taxonomy database (Sayers et al., 2008); note that orders are not always monophyletic. Support of bipartitions in the consensus tree is shown by node color. Branch lengths indicate the average number of substitutions per site; see scale bar at the bottom. “Multiple taxa” in Chroococcidiopsidales stands in for closely related taxa whose ScyB sequences are identical to each other.

The ScyB phylogeny (Figure 3) shows Clade 9 as basal to all other included Cyanobacteria, as expected from the previous species trees and the 16S tree. However, it also groups *Chroococcidiopsis* outside Clade 10 in a more basal part of the tree together with a clade of unclassified cyanobacteria, albeit with relatively low support (59%). This departure from the species tree is also the feature of note on the automated protein tree‐species tree reconciliation (Figure S2 for ScyB): it invokes a transfer into the Clade 11 taxa to explain their more derived placement compared with *Chroococcidiopsis*, with other identified events being shallow. Finally, the divergent sequences from non‐scytonemin‐producing *Cylindrospermopsis* and *Raphidiopsis* group together with *Cyanothece* sp. PCC 7424 and PCC 7822 with a posterior probability of 60%, and the very long branch further casts doubt on this placement. In general, the ScyB tree closely follows the ScyC tree with high support values, showing a shared phylogenetic history between the two proteins.

### 3.3 ScyA phylogeny

**FIGURE 4:**
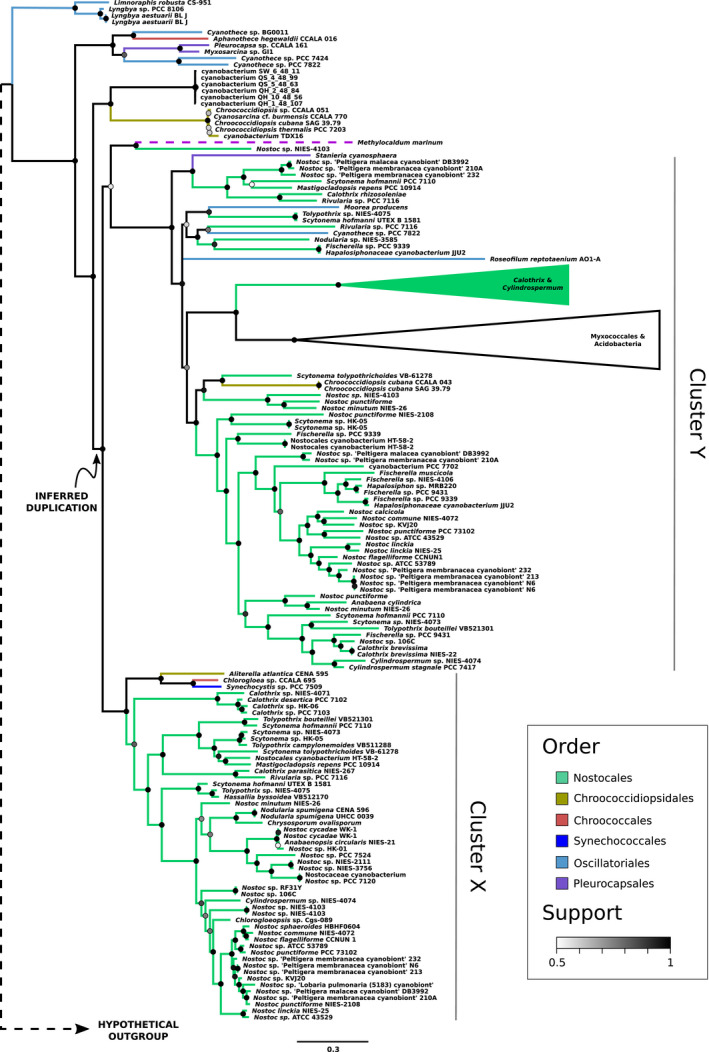
Bayesian phylogenetic tree based on scytonemin biosynthesis protein ScyA sequences. Branches are colored according to taxonomic order as provided by the NCBI taxonomy database (Sayers et al., 2008); note that orders are not always monophyletic. Support of bipartitions in the consensus tree is shown by node color. Branch lengths indicate the average number of substitutions per site; see scale bar at the bottom. A marked duplication event is inferred to be responsible for two clusters of ScyA copies in the derived part of the tree (see text). The placement of *M. marinum* is shown with a broken purple line to indicate uncertain placement.

Phylogenetic analysis of ScyA presents a more complicated tree than ScyC and ScyB: it contains two cyanobacterial clusters sister to each other and with many of the same taxa (labelled X and Y, see Figure 4) as well as some earlier‐branching lineages. Cluster X contains Nostocales with close relatives and largely recapitulates their relationships from ScyC and ScyB trees. Cluster Y is dominated by the same taxa, but also contains some additional groups within Nostocales such as *Fischerella*, missing from cluster X and other Scy protein trees.

This pattern is best explained by an ancient duplication of the gene encoding ScyA, pre‐dating the last common ancestor of Nostocales but likely post‐dating the divergence of Clade 10 from other lineages. Earlier‐branching lineages appear basally in the tree and its members tend to carry only one copy of ScyA. Overall, one copy has been retained in all scytonemin producers—including cluster X in Nostocales and close relatives—reflecting the same pattern of retention and vertical inheritance as ScyC and ScyB. Perhaps functionally divergent, the other copy—cluster Y in Nostocales—has been under less purifying selection, as evidenced by more evolutionary change (longer branches) and further recent duplications. Unfortunately, the inference of this ancient duplication could not be tested by the automated protein tree‐species tree reconciliation, which failed to find any temporally feasible solution, likely due to the increased complexity of the ScyA tree together with the inherent phylogenetic uncertainty in the reconstruction.

Also embedded in cluster Y are two groups of divergent sequences, one containing members of genera *Calothrix* and *Cylindrospermum* and the other members of Myxococcales and Acidobacteria. Of the sequences in the tree, these bear the least similarity to the query and were included by the conservative cutoff in order not to exclude other sequences relevant to the study (see “Sequence collection” above). Their placement on long branches indeed reflects a distant relationship with the main cluster of included sequences, but the placement of each group within cluster Y is robust to the exclusion of its sister (Figures S3 and S4), suggesting that any misplacement is not a product of long branch attraction.

### 3.4 Rooting

While sequences related to each Scy protein are present in non‐cyanobacterial *M. marinum*, we do not know a priori how these sequences relate to the Scy protein copies in Cyanobacteria. Thus, we cannot be entirely confident in treating *M. marinum* as an outgroup to the cyanobacterial ingroup. However, this proposed rooting is strongly suggested by the Scy trees closely resembling a species tree, as alternative rootings would force non‐parsimonious gene histories.

The observed duplication in ScyA also constrains the position of the root, using reciprocal rooting between paralogs (Gogarten et al., 1989). This is independent of arguments regarding species tree congruence that support the proposed rooting of all Scy protein trees. Reciprocal rooting of clusters X and Y constrains the root of the tree to be basal to both, consistent with the root suggested by the species tree. Note that, for ScyA, the long branch leading to putative outgroup *M. marinum* falls to a basal position inside cluster Y with moderate support (55%), perhaps an unreliable placement among other long branches in this part of the tree. In fact, with a slightly different taxon sampling it moves basal to Cluster X (Figure S3).

The inferred vertical inheritance of Scy proteins suggests that scytonemin production was present by the last common ancestor of Clade 10, Clade 11, and Clade 9. Although *Lyngbya* and *Limnoraphis* of Clade 9 are not always basal to other Cyanobacteria as on the species tree, this is explained by relatively small movements of the root position across adjoining branches—well within the rooting uncertainty without a close outgroup. The basal part of the tree also contains sparsely populated long branches, and long branch attraction artifacts are quite possible. Thus, a vertical inheritance of scytonemin production in *Lyngbya* and *Limnoraphis* remains a more plausible and simpler explanation than a more recent acquisition via gene transfer from other basal lineages on the tree.

Scytonemin production in *Lyngbya aestuarii*, among the most deeply branching taxa on the rooted tree, has also been confirmed experimentally (Balskus et al., 2011). This helps establish that the recovered Scy trees span functional versions of Scy proteins, even though the functionality has not been experimentally demonstrated in every individual taxon.

### 3.5 Dating the origin of scytonemin

These Scy protein phylogenies indicate that the scytonemin clade (defined by production of this pigment as a shared derived character) includes all descendants of the first scytonemin‐capable common ancestor of Clade 10, Clade 11, and Clade 9. How old is this clade and does the beginning of scytonemin production correlate with known atmospheric or planetary changes in Earth history? The congruence of Scy protein trees to published species trees of Cyanobacteria permit divergence time estimates from the latter to be mapped to the former.

However, existing age estimates for events in cyanobacterial evolution vary substantially based on dataset, clock model, and fossil calibration choices (Sánchez‐Baracaldo, 2015; Schirrmeister et al., 2011). For example, Schirrmeister et al. (2011) test a number of different calibration hypotheses resulting in a broad range of possible origin dates for scytonemin. Given these age estimates, at its youngest, the last common ancestor of Clade 9, Clade 10, and Clade 11 falls at 2.05 Ga. The earliest divergence of this lineage from its sister falls at 2.95 Ga, establishing an older age bound for scytonemin production within cyanobacteria. End‐member calibration approaches thus present an almost billion‐year possible range for the origin of scytonemin and correlating its timing with planetary events requires a reduction in this uncertainty.

More recently, cyanobacterial divergence times have been estimated using a set of horizontal gene transfer events that impose novel constraints on both posterior age estimates and evolutionary model choice (Fournier et al., 2021). Using a representative set of fossil calibrations in Cyanobacteria, this work produces species tree divergence time estimates indicating that scytonemin appears between 2.18 and 2.03 Ga (older bound on the stem age to younger bound on the crown age of the scytonemin clade). Thus, while still based on an interpretation of the fossil record and including uncertainties from evolutionary rate estimation, this narrower range more strongly suggests that scytonemin appeared after the GOE. The estimate also falls well within the broad range of 1.7–3.0 Ga previously obtained from an analysis of auxiliary scytonemin biosynthesis protein trees (Garcia‐Pichel et al., 2019).

## 4 DISCUSSION

Phylogenies of scytonemin biosynthesis enzymes and molecular clock studies of Cyanobacteria indicate that scytonemin production evolved in the Paleoproterozoic, after the GOE and the establishment of the ozone layer. This suggests that cyanobacteria only adapted to become more resistant to UVA after a UV‐protective atmosphere was present. Thus, the threat of UVA is curiously inverted compared to UVB and UVC: once the surface flux of short‐wavelength UV wanes and perhaps allows for easier growth in exposed environments, UVA becomes a threat and its abatement comes under a stronger selective pressure (see Table 2 for a synopsis of the time‐varying relationship between oxygen, UV, and scytonemin).

**TABLE 2:** Synopsis of UV conditions and scytonemin response through Earth history

	Archaean	Proterozoic	Modern
Oxygen/ozone	None	Below present	Present levels
UV	UVC and UVB, *UVA not a threat*	No UVC, less UVB, *UVA now a threat*	UVA, some UVB
Scytonemin	None	Becomes important	Remains

It is entirely possible that mechanisms were in place to protect cyanobacteria from short‐wavelength UV radiation even before the GOE: for example, mycosporine‐like amino acids may have been in use for protection against UVB already, as their broad taxonomic distribution (Sinha et al., 2007) reflects an ancient origin. UVB and UVC are also attenuated significantly better in water than UVA (Cockell, 2000): an organism at manageable depth with respect to UVB and UVC could still experience significant UVA irradiation. Also, importantly, UVA would become increasingly harmful as oxygen became more available across different environments. Therefore, ironically, some cyanobacteria may have experienced increased radiation damage from UV following the GOE.

Even if Archean atmospheric oxygen levels had not yet accumulated to a sufficient extent to induce widespread damage to cells via UVA radiation, cyanobacterial mats may have experienced localized oxygen concentrations high enough to be harmful in this regard. Such a scenario could even have selected for microbial mat ecologies to include mutualistic bacteria performing aerobic respiration, which would prevent the accumulation of oxygen to harmful levels. This has previously been proposed with regard to mitigating oxidative stress induced by cellular processes (Taverne et al., 2020), but the additional oxidative stress induced by UVA radiation may have posed an even greater selective pressure.

In today's ocean water, about 10% of incident UVA radiation penetrates to more than 10 m of depth (Tedetti & Sempéré, 2006). In the absence of plant‐derived organic material in the Archaean, this penetration was likely much greater (Mloszewska et al., 2018), whether in the sea or freshwater environments. Organisms living in the shallow ocean, intertidal zone, or in freshwater bodies would have been subject to more intense UVA than today. High penetration of UVA would have applied considerable evolutionary pressure to develop a protective mechanism such as scytonemin, promptly upon the introduction of oxygen to the atmosphere and surface waters. Furthermore, any cyanobacteria living subaerially at the time would not have benefitted from any protection by a layer of water. Independently of any molecular clock, we should expect that the appearance of scytonemin be tied closely to the GOE.

There are other reasons to anticipate the appearance of scytonemin not just after the GOE, but soon after. Some geochemical evidence suggests that while oxygen levels were generally higher in the Proterozoic than before the GOE, they first hit a peak in the immediate aftermath of the GOE and then decreased again, forming an “overshoot” overlapping with the Lomagundi carbon isotope excursion and subsiding with it before 2.0 Ga (Partin et al., 2013). In this scenario, oxidative stress and the evolutionary pressure to develop UVA protection mechanisms would have been highest in the immediate aftermath of the GOE, decreasing later. Potential further need for UV protection during the early Paleoproterozoic derives from large‐scale glaciations (Kopp et al., 2005), increasing irradiation via surface albedo and prompting high photoprotective pigment responses such as documented for scytonemin in the modern Arctic (Quesada et al., 1999). Furthermore, it is possible that persistently cold conditions would have slowed the biochemical repair processes and increased reliance on the photoprotective response instead. However, this particular scenario is conditional on greater environmental uncertainties than the more certain increase in oxidative stress due to higher oxygen levels.

In any case, this perspective also suggests that scytonemin appearance could be used as a time calibration on cyanobacterial evolution, much like has been proposed previously for heterocyst development (Schirrmeister et al., 2011)—also an adaptation to increased oxygen levels. For both traits, we can assume that wherever they appear on the tree should post‐date the GOE. Furthermore, an expectation that scytonemin would have appeared soon after the GOE (by, say, 2.0 Ga as per the discussion above) also offers a younger bound in addition to the older one. This additional constraint pushes Cyanobacteria basal to the scytonemin clade further into the past and the origin of crown Cyanobacteria well before the GOE. Thus, the scytonemin phylogenies, considered independently of molecular clocks, offer further evidence that crown Cyanobacteria appeared well before the GOE (Schirrmeister et al., 2011, as well as others) and not after (Shih et al., 2017).

It is notable that we found no evidence of any group acquiring the capacity for scytonemin production by horizontal gene transfer, otherwise a common process facilitating adaptive radiations in the microbial world. Widespread acquisition of scytonemin production might have been hindered by the relatively complex genetic machinery involved, though a transfer of the complete scytonemin operon seems feasible. Alternatively, the consignment of scytonemin to a particular clade of Cyanobacteria might reflect unique UVA pressures they face, growing in highly exposed environments with the presence of oxygen.

Multiple independent losses of the genetic ability to produce scytonemin are inferred within the scytonemin clade; however, any ecological pattern related to differential UVA exposure that explains the observed retentions and losses of scytonemin synthesis is not obvious. Both loss and retention seem to have occurred widely whether the cyanobacteria in question have unicellular or multicellular lifestyles and whether they live in fresh water or the sea—or under a layer of rock, as is the case for *Chroococcidiopsis* that have retained scytonemin in that environment. Some relatively shallow losses after billions of years of unbroken vertical inheritance might be explained by the relatively stable oxygen concentrations over the Phanerozoic (Lyons et al., 2014): since scytonemin would be the most useful upon an increase in oxygen levels and UVA‐induced oxidative stress, it is possible that an important bottleneck selecting for the presence of scytonemin occurred during the Neoproterozoic Oxygenation Event and the associated Cryogenian glaciations. After that, as other mechanisms that neutralize reactive oxygen species—such as enzymatic breakdown and scavenging (He et al., 2017; Xie et al., 2009)—were honed to cope with Phanerozoic oxygen concentrations, many organisms may not have suffered in fitness by losing the expense of scytonemin production.

Regardless of sporadic losses, this phylogenetic evidence reflects a general vertical inheritance of scytonemin production within Cyanobacteria, with an origin during a time of major redox transition in Earth history following the GOE. This pattern reflects a persistent need for protection from UVA in an oxygenated world.

## CONFLICT OF INTEREST

The authors declare that there is no conflict of interest.

## Supporting information


**Appendix S1** Supporting Information

## Data Availability

The data associated with this study, including multiple sequence alignments and tree files, are available in Dryad (https://doi.org/10.5061/dryad.w6m905qq6).
