# Supplementary material for: Inferred ancestry of scytonemin biosynthesis proteins in cyanobacteria indicates a response to Paleoproterozoic oxygenation
Source: Geobiology. 2022 Jul 18;20(6):764–75. doi: 10.1111/gbi.12514 (PMC9796282; doi:10.1111/gbi.12514)
Supplement: Supplementary file 1 — Appendix S1 Supporting Information [file GBI-20-764-s001.docx]

# **Supplementary Material**

### **Protein tree-species tree reconciliation with Notung**

Results of protein tree-species tree reconciliation are shown in Figures S1 and S2 for ScyC and ScyB, respectively; there were no temporally feasible solutions recovered for ScyA. See Methods in the main text for the parameters used in the reconciliation.

### **ScyA tree long-branch attraction tests**

Among Cluster Y on the ScyA tree (Figure 4 in the main text) are two groups of divergent sequences, one containing members of genera *Calothrix* and *Cylindrospermum* and the other members of Myxococcales and Acidobacteria. Since these clusters of sequences on long branches placed sister to each other in the tree, we tested whether this placement might be a long-branch attraction (LBA) artifact. We made two trees, each with sequences belonging to one of the groups removed, to see if the placement of the other group would change (Figures S3 and S4).

In both cases, the group still in the tree retained its previous placement within Cluster Y, suggesting that the placement of each group is not a product of long-branch attraction.

Note that the placement of *Methylocaldum marinum* also varies between the two trees. While S2 shows it as basal to Cluster Y like the original ScyA tree, in S1 it moves basal to Cluster X – with relatively low support in each case. This is consistent with an unreliable placement of *Methylocaldum marinum* in the complex ScyA tree, as discussed in the main text.


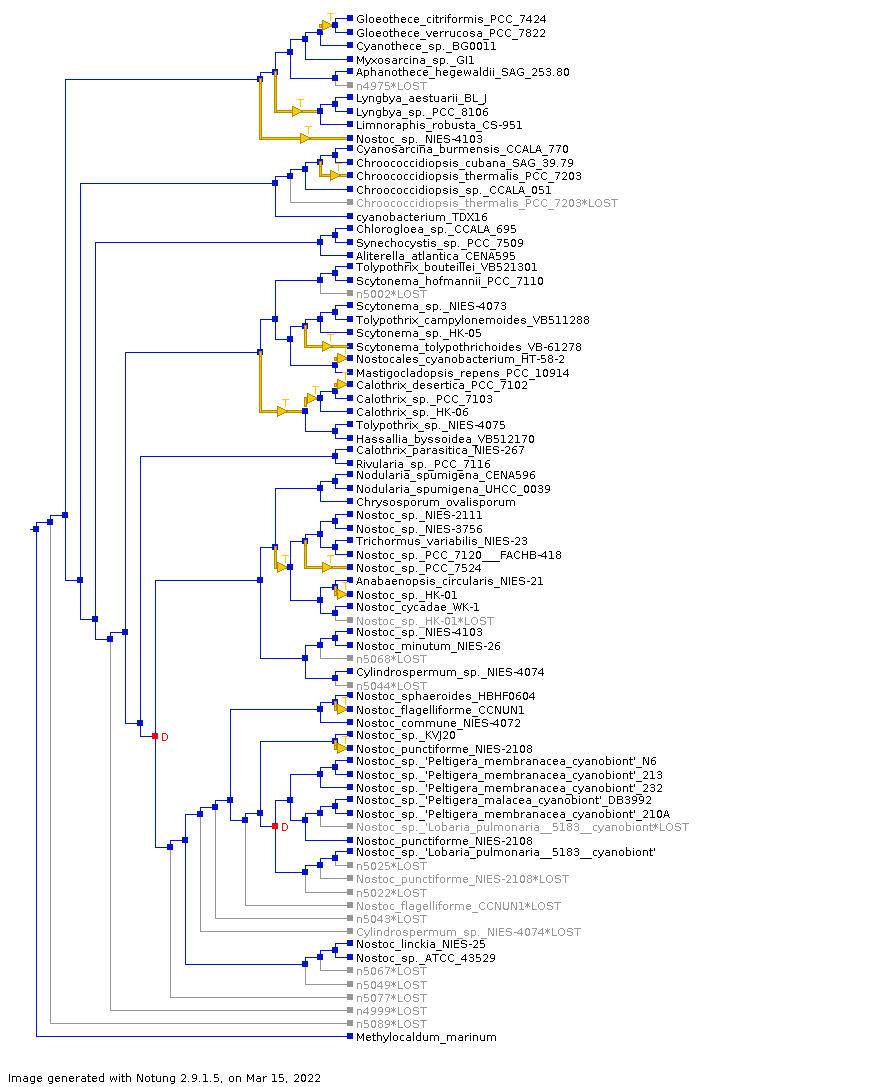
Figure S1. Protein tree-species tree reconciliation for ScyC: red dots signify inferred duplications and yellow arrows transfers, while grey lines show losses.


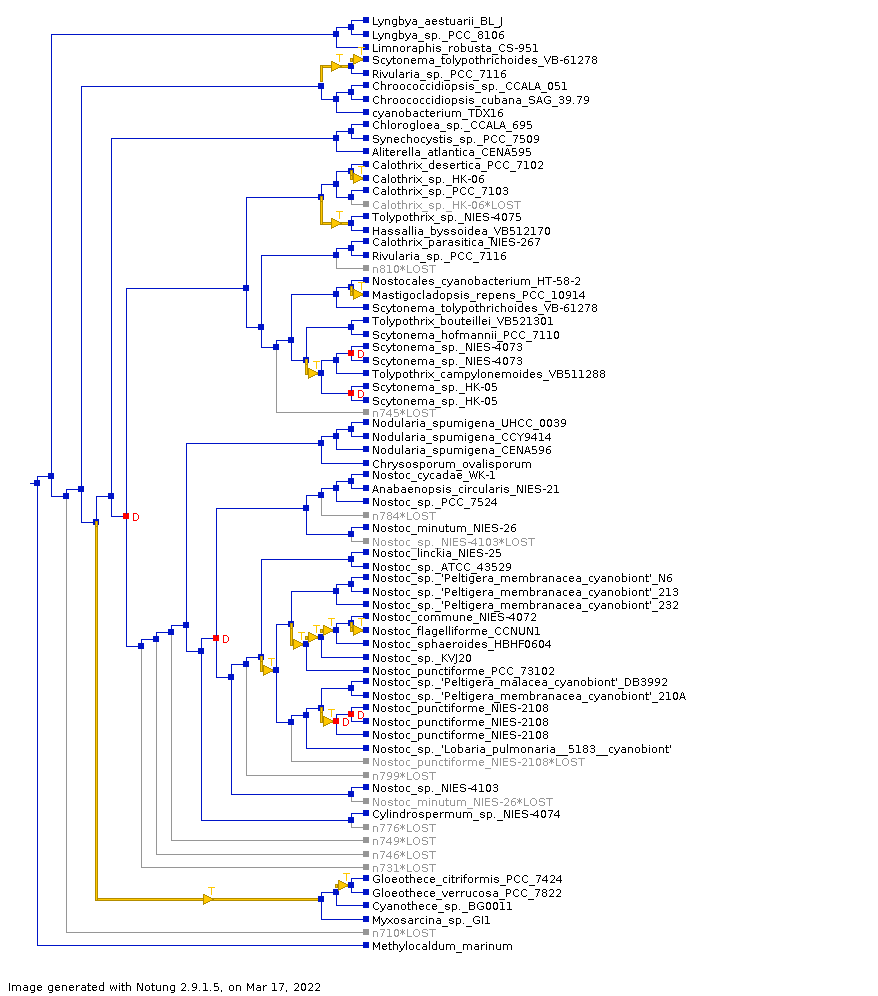

Figure S2. Protein tree-species tree reconciliation for ScyB: red dots signify inferred duplications and yellow arrows transfers, while grey lines show losses.


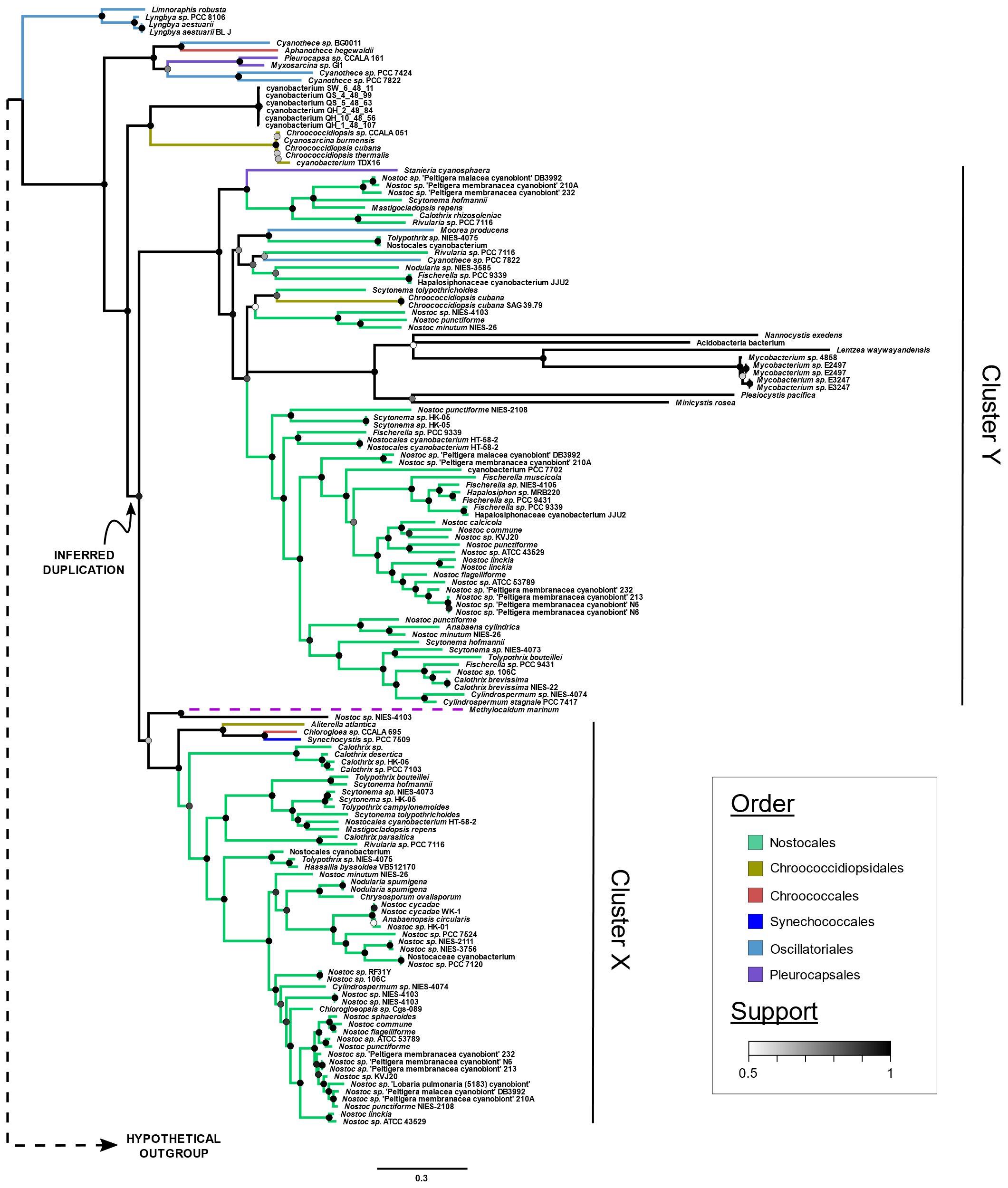


Figure S3. A Bayesian phylogenetic tree based on scytonemin biosynthesis protein ScyA copies, with a group of divergent sequences containing members of genera Calothrix and Cylindrospermum removed to test for long-branch attraction with its sister group. Branches are colored according to taxonomic order as provided by the NCBI taxonomy database [50]; note that orders are not always monophyletic. Support of bipartitions in the consensus tree is shown by node color. Branch lengths reflect the average number of substitutions per site; see scale bar at the bottom. A marked duplication event is inferred to be responsible for two clusters of ScyA copies in the derived part of the tree (see main text). The placement of Methylocaldum marinum is shown with a broken purple line to indicate uncertain placement.


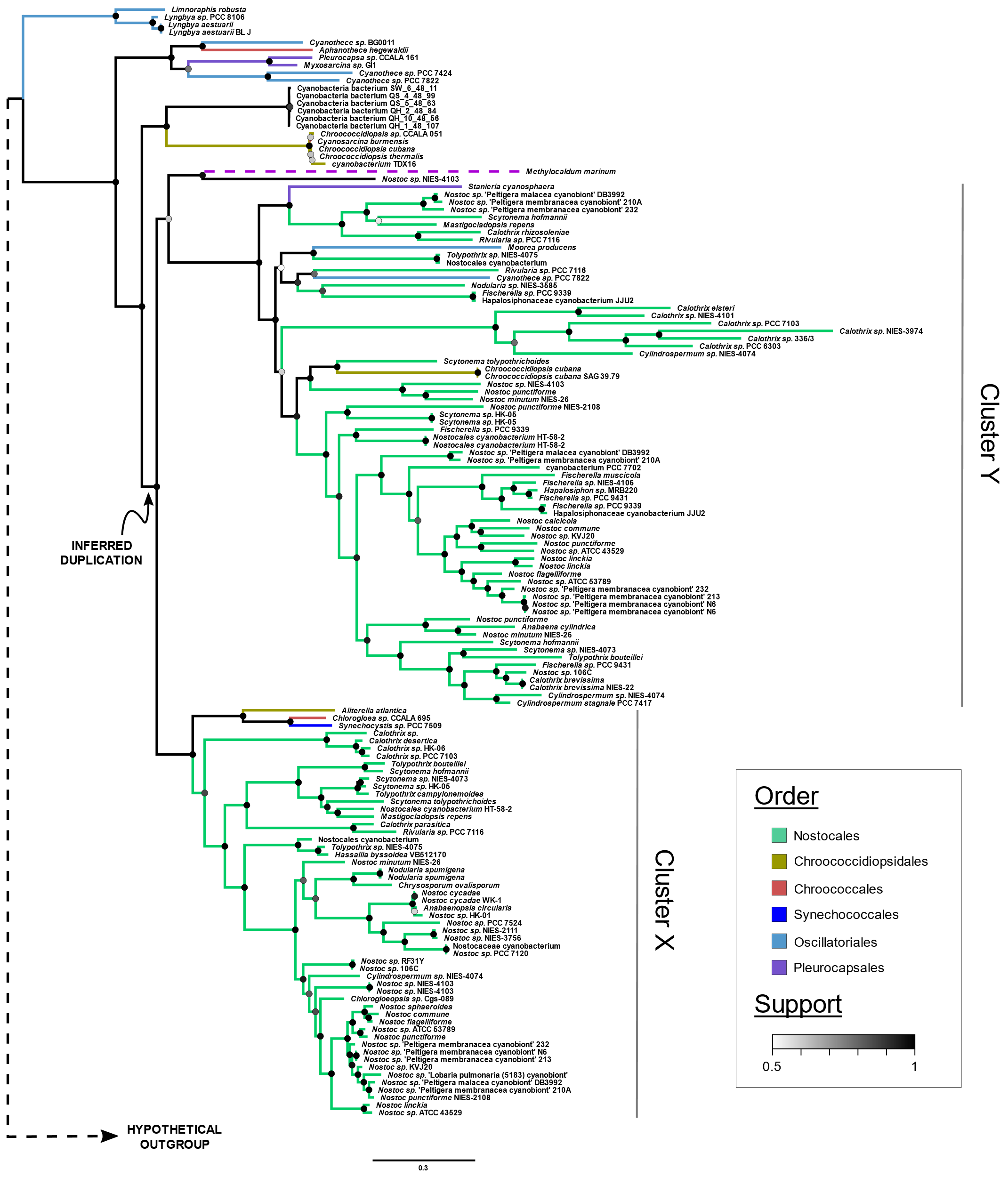


Figure S4. A Bayesian phylogenetic tree based on scytonemin biosynthesis protein ScyA copies, with a group of divergent sequences containing members of Myxococcales and Acidobacteria removed to test for long-branch attraction with its sister group. Branches are colored according to taxonomic order as provided by the NCBI taxonomy database [50]; note that orders are not always monophyletic. Support of bipartitions in the consensus tree is shown by node color. Branch lengths reflect the average number of substitutions per site; see scale bar at the bottom. A marked duplication event is inferred to be responsible for two clusters of ScyA copies in the derived part of the tree (see main text). The placement of Methylocaldum marinum is shown with a broken purple line to indicate uncertain placement.
